# Evaluation of Fusarium Head Blight Resistance Effects by Haplotype-Based Genome-Wide Association Study in Winter Wheat Lines Derived by Marker Backcrossing Approach

**DOI:** 10.3390/ijms232214233

**Published:** 2022-11-17

**Authors:** Magdalena Radecka-Janusik, Urszula Piechota, Dominika Piaskowska, Tomasz Góral, Paweł Czembor

**Affiliations:** Plant Breeding and Acclimatization Institute—National Research Institute, Department of Applied Biology, Radzików, 05-870 Błonie, Poland

**Keywords:** *Triticum aestivum*, *Fusarium culmorum*, marker-assisted selection, GWAS, association weight matrix

## Abstract

Fusarium head blight (FHB) of wheat caused by *Fusarium* species is a destructive disease, causing grain yield and quality losses. Developing FHB-resistant cultivars is crucial to minimize the extent of the disease. The first objective of this study was incorporation of *Fhb1* from a resistant donor into five Polish wheat breeding lines with good agronomical traits and different origins. We also performed a haplotype-based GWAS to identify chromosome regions in derived wheat families associated with Fusarium head blight resistance. As a result of marker-assisted backcrossing (MABC), five wheat combinations were obtained. Fungal inoculation and disease assessment were conducted for two years, 2019 and 2020. In 2019 the average phenotypic response of type II resistance was 2.2, whereas in 2020 it was 2.1. A haploblock-based GWAS performed on 10 phenotypic traits (related to type of resistance, year of experiment and FHB index) revealed nine marker–trait associations (MTA), among which six belong to chromosome 2D, two to 3B and one to 7D. Phenotypic variation (R^2^) explained by the identified haplotypes in haploblocks ranged from 6% to 49%. Additionally, an association weight matrix (AWM) was created, giving the partial correlation–information theory (PCIT) pipeline of 171 edges and 19 nodes. The resultant data and high level of explained phenotypic variance of MTA create the opportunity for data utilization in MAS.

## 1. Introduction

Fusarium head blight (FHB) is caused by *Fusarium* species, mainly *F. culmorum* and *F. graminearum* [1]. FHB occurs in all wheat production areas and has received growing attention over the past decades. Under favorable conditions, with increasing humidity and warm temperatures, it may lead to severe grain quality and yield losses. Moreover, it is known that mycotoxin accumulation in grain endangers food and feed safety as FHB pathogens contaminate grain with trichothecenes and other fungal metabolites that pose a health risk to humans and animals [2,3,4,5].

Controlling the disease through agronomic practices, such as crop rotation and straw retention, as well as fungicide use, is only partly effective. Developing FHB-resistant cultivars is crucial to minimize losses caused by the disease, which may reach epidemic proportions, develop moderately or remain low and undetected [6]. Thus, breeding for FHB resistance has become an important goal for cereal breeders as it significantly reduces the need for fungicide application. It is an economically and environmentally effective strategy for controlling FHB. The overarching goal in resistance breeding is the development of cultivars characterized by high yield with few disease symptoms and low mycotoxin contamination despite high infection pressure. Of great importance is production of regionally adapted cultivars with a good level of FHB resistance. There are a few wheat germplasm sources that can be used as resistance donors. Asian cultivars are mainly used as resistant or moderately resistant sources for breeders [7]. Among them, Sumai3 (and derivatives), a spring wheat cultivar, is one of the most important and best characterized sources of FHB resistance [8]. This cultivar incorporates several resistance effective quantitative trait loci (QTL) [9].

Fusarium head blight resistance is quantitative, influenced by environmental factors, with significant genotype–environment interactions. Resistance to FHB has been categorized into five types: type I for resistance to initial infection by the pathogen and type II for resistance to fungal spread along the rachis were first described by Schroeder and Christensen [10]; other types of resistance to FHB were described by Mesterhazy [11] and Mesterhazy et al. [12]. More than 500 QTL for resistance explaining different phenotypic variations have been reported thus far [6,9,13]. Most known QTL contributing to the trait have a small to medium effect, which is further influenced by the environment [14]. The best characterized QTL are *Fhb1* (syn. *Qfhs.ndsu-3BS*), with the main effect on chromosome 3BS, and *Qfhs.ifa-5A*, with the main effect on chromosome 5A [14].

The most stable type II resistance QTL, *Fhb1*, is the best characterized FHB locus with a major effect. *Fhb1* confers resistance to fungal spread and to toxin accumulation [14]. This locus explains 20% to 60% of the phenotypic variation [15] and has been widely integrated into wheat breeding populations through marker-assisted selection (MAS) [9,14,16]. It was reported that depending on the genetic background, the disease severity can be reduced to various levels [17]. Diagnostic markers in the *Fhb1* region have been developed and are useful for MAS, enabling easier transfer of *Fhb1* into cultivars with the desired characteristics [15,18,19,20,21]. However, recent studies revealed that the nature of *Fhb1* resistance remains unclear [22].

Various methods can be used to introduce the resistance gene into varieties or lines. In the present research, marker-assisted backcrossing (MABC) was used to incorporate resistance QTL for FHB resistance, as performed in other studies [23,24]. However, using doubled haploid (DH) lines after crossing with a resistance source [25] and recombinant inbred lines (RILs) [26] is also recommended and widely practiced for breeding for FHB resistance.

Association mapping in wheat based on linkage disequilibrium (LD) is a novel and powerful approach to identify the relatedness between molecular markers and FHB resistance in wheat lines derived by MABC [27,28]. Based on the scale of the research, association mapping can be classified as genome-wide association studies (GWAS) [29] which, using a high-throughput genotyping tool, such as single nucleotide polymorphism (SNP) arrays, generates reliable results. Markers identified in GWAS can be used for MAS directly or after conversion to utility markers [30,31]. Several GWAS for FHB resistance, which confirm its complex architecture, have been reported [32,33,34,35,36]. In our work, we also chose to apply GWAS for better prospects. Therefore, the goals of the study were (i) to incorporate *Fhb1* from a resistant donor into five Polish wheat breeding lines with good agronomical traits and different origins and (ii) to conduct haplotype-based GWAS to identify chromosome regions in derived wheat families associated with Fusarium head blight resistance.

## 2. Results

### 2.1. Fusarium Head Blight Response Lines

The FHB response was evaluated under polytunnel conditions on an experimental field at one site in Poland over two consecutive years, 2019 and 2020 (Table S1). In response to artificial inoculation with two *F. culmorum* isolates, the reaction of five groups of wheat cultivars and lines was compared. Disease symptoms for type II resistance were moderate to high. In 2019 the average phenotypic response of type II resistance was 2.2 with a minimum of 0.6 and maximum of 5.8. In 2020, the average phenotypic response of type II was 2.1, while the minimum was 0.9 and the maximum was 6.2. The FHB index (FHBi) ranged from 10 to 100% (susceptible check KBP 14 16 (S)) with an average value of 54%, but due to unfavorable weather conditions, FHBi data were available only for the year 2019.

**Figure 1:**
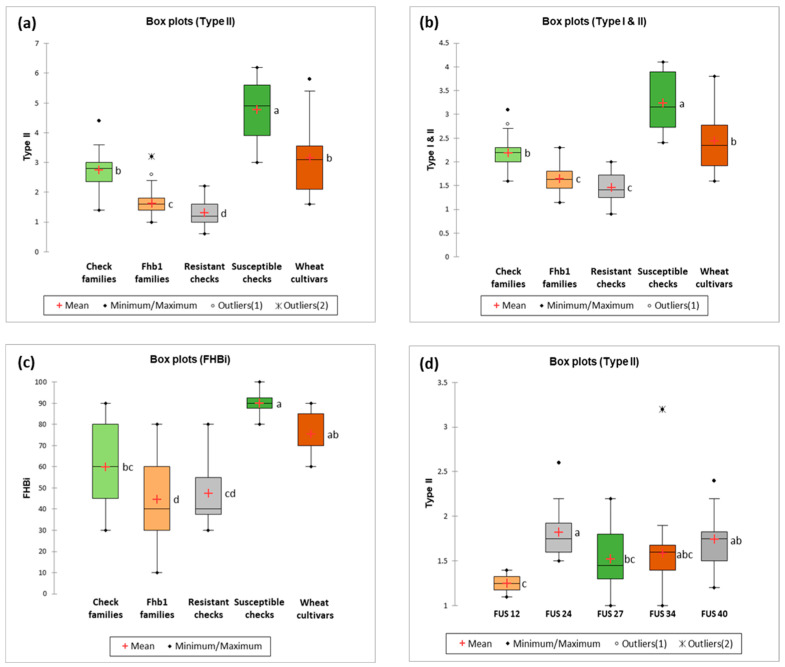
(**a**) Box plot of type II resistance to Fusarium head blight of wheat groups (years 2019 and 2020); boxes marked with the same letter are not significantly different at *p* < 0.05 according to Tukey’s HSD test performed on log10 transformed data. (**b**) Box plot of mixed type I & II resistance to Fusarium head blight of wheat groups (years 2019 and 2020); boxes marked with the same letter are not significantly different at *p* < 0.05 according to Tukey’s HSD test performed on log10 transformed data. (**c**) Box plot of Fusarium head blight index of wheat groups; boxes marked with the same letter are not significantly different at *p* < 0.05 according to Tukey’s HSD test performed on Box–Cox transformed data. (**d**) Box plot of type II resistance to Fusarium head blight of families of five wheat combinations (years 2019 and 2020); boxes marked with the same letter are not significantly different at *p* < 0.05 according to Tukey’s HSD test performed on log10 transformed data.

In the case of type I FHB resistance (not shown in the box plot), there were no significant differences between wheat groups. Type II FHB resistance showed significant differences between all pairs of groups except check families and wheat cultivars. Fhb1 families derived in our work significantly differed from all wheat groups (Figure 1a). Type I and II average FHB resistance was very similar to type II FHB resistance. The wheat groups differed in phenotypic response, but between check families and wheat cultivars, as well as between Fhb1 families and resistant check, the differences were not significant (Figure 1b). As regards the FHB index, all groups were pairwise compared with Tukey’s test. It was found that Fhb1 families significantly differed in the level of infection from check families, susceptible checks and wheat cultivars (Figure 1c). Comparison of the five tested combinations of Fhb1 families was also performed (Figure 1d). It was noted that families FUS_12 and FUS_24 significantly differed between each other, while the remaining families displayed a similar level of phenotypic response.

### 2.2. SSR Marker-Based Selection

At each step of molecular analysis, a different number of selected plants was obtained. After MAS of the F_1_BC_1_ generation of all five combinations with central and flanking markers, 53 plants that possessed the desired alleles were selected. The obtained F_1_BC_2_ generation after molecular selection revealed 62 desirable plants from all combinations. Next, the F_2_BC_2_ generation was obtained and after MAS, 55 plants were chosen to produce F_3_BC_2_ families. From those plants, 35 were selected for genotyping on the DArTseq platform (Diversity Arrays Technology P/L, Bruce, Australia).

### 2.3. Population Structure Analysis and Haploblock Calling

**Figure 2:**
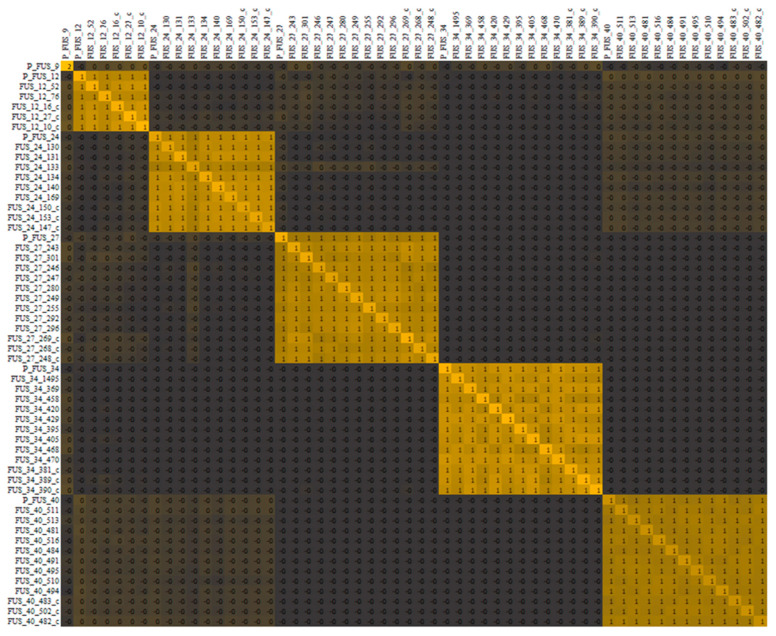
Heatmap of cluster analysis using kinship similarity matrix of 58 wheat genotypes based on DArT-SNP markers.

A total of 23,788 DArT-SNP markers were generated using the DArTseq platform. After filtering steps, 10,251 informative DArT-SNP markers were used for further studies. Similarity analysis employing a kinship matrix revealed five clusters which were consistent with wheat families (Figure 2).

**Figure 3:**
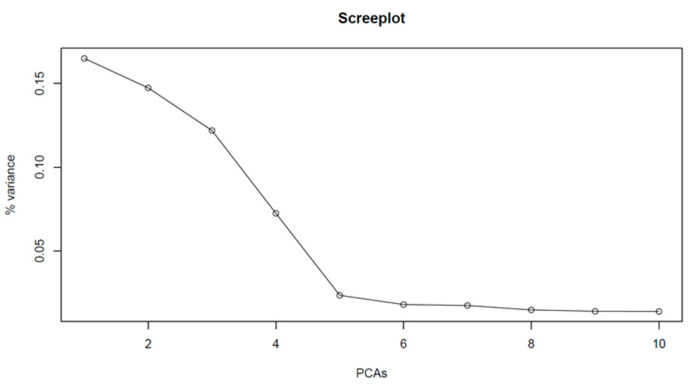
Principal component analysis on the 2256 haploblocks of 58 wheat genotypes. Scree plot of the percentage of the variance explained by the first 10 PCs.

In total, 2256 HBs were identified consisting of 8373 HTs. Based on these data, PCA analysis was conducted (Figure 3).

Detailed data showed that genome A consisted of 706 HBs. Genome B was built of 803 HBs and the lowest number of HBs was identified on genome D (Table 1).

**Figure 4:**
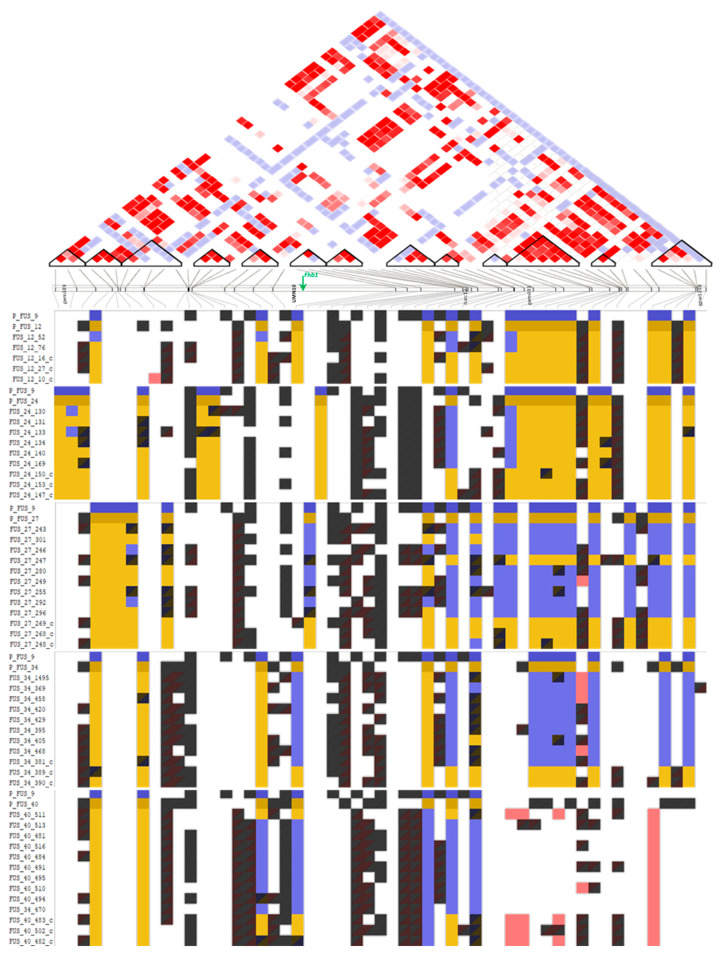
Schematic representation of LD plot and haploblocks (black triangles) detected on 3BS chromosome fragment (0.7–23.1 Mb) with estimated SSRs marker (black) and *Fhb1* locus (green arrow) location; the red color indicates the high level of LD. Below the DArT-SNP allele distribution in five wheat families and parental lines; the blue indicates the resistant parent allele, the yellow part correlates with the RP allele; pink indicates a novel allele not represented in parental lines; the remaining one indicates a common allele for both parental lines.

On average, the number of SNP markers per HB ranged from 3.1 on chromosome 4D to 5.2 on chromosome 2B, while the maximum was 58 SNPs (chromosome 2B). The mean HB ranged from 1.8–3.8 Mb on genome D to 2.6–4.9 Mb on genome A. The largest one was constructed on chromosome 5A (115.1 Mb). The mean chromosome coverage reached 50.1% on genome B and a little less on genomes A and D (49.8 and 49.1, respectively). Visualization of HBs and HTs located within *Fhb1* flanking markers is shown in Figure 4.

### 2.4. GWAS Analysis

GWAS analysis was based on 8373 haplotypes from 2256 haploblocks. Using Bonferroni correction (−log(*p*) > 5.22), there were nine marker–trait associations (MTA) identified, among which six belong to chromosome 2D, two to 3B and one to 7D (Table 2).

**Figure 5:**
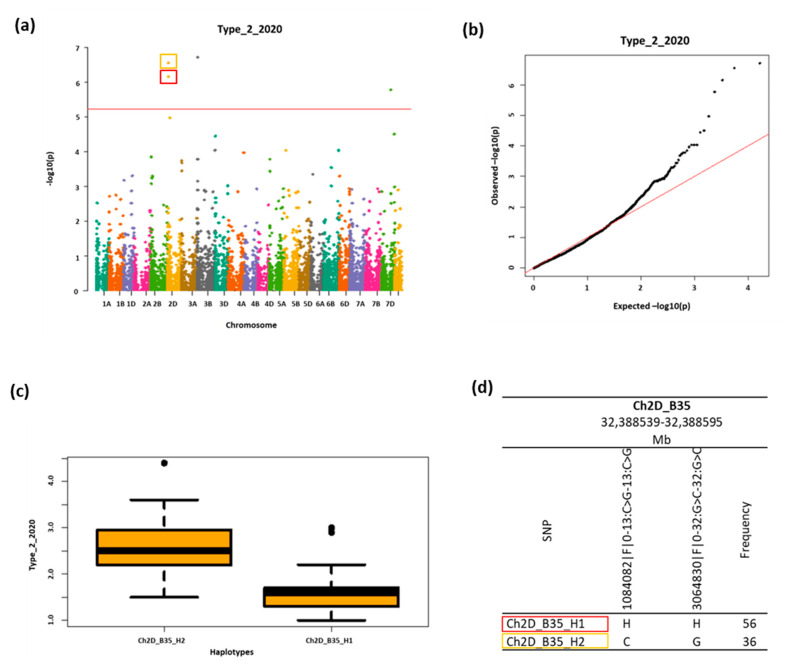
(**a**) Manhattan plot on 21 wheat chromosomes for Type_2_2020 trait. The red horizontal dash represents the GWAS threshold of −log(*p*) = 5.22 for Bonferroni correction and dots above the red line indicate significant MTAs. (**b**) Q-Q plot for GWAS results for Type_2_2020 trait. (**c**) Boxplots indicating the phenotype value of Type_2_2020 trait corresponding to the two haplotypes (H1 and H2) in the same haploblock (Ch2D_B35) on chromosome 2D. (**d**) Haplotype block based on two SNP markers on chromosome 2D; both haplotypes are significantly associated with the Type_2_2020 trait (MTA). Frequency show occurrence (percentage) of haplotypes in the group of families.

Two haplotypes located on chromosome 2D significantly associated with four traits (Table 2). Haplotype Ch2D_B35_H1 was linked to traits Type_2_2020 (an example is shown in Figure 5) for type II resistance to FHB, Type_2_2019&2020 (Figure S1) for average type II resistance to FHB from two years (2019 and 2020) and with the FHB index (Figure S2). Another haplotype on 2D (Ch2D_B35_H2) was mapped in the same location and collocated with Type_2_2020 (Figure 5) and Type_2_2019&2020 (Figure S1) (as above). Additionally, this HT associated with the Type_1&2_2020 (Figure S3) trait (average for type I and type II resistance to FHB in 2020). Those haplotypes belong to the same haploblock, which means the same locus, but HTs are represented in different lines (Table S2). HT Ch3B_B9_H2 on chromosome 3B significantly associated with two traits: Type_2_2020 (Figure S4) and Type_2_2019&2020 (Figure S5), similar to HTs on chromosome 2D. Type_2_2020 for type II resistance to the FHB trait was also linked to HT Ch7D_B63_H4 on chromosome 7D (Figure S6). Phenotypic variation (R^2^) explained by the identified haplotypes in haploblocks ranged from 6% for Ch2D_B35_H1 to 49% for Ch3B_B9_H2 (Table 2).

### 2.5. Fhb1 Locus

Analysis of the *Fhb1* gene location on chromosome 3BS covered a fragment which was flanked by markers associated with this gene and spanned 0.7–23.1 Mb. Haploblock calling showed 13 HBs on this fragment (Figure 4). The *Fhb1* locus was not covered by DArT-SNP markers for calculated HBs. The DArT-SNP allele mining within analyzed families indicated FUS_24, FUS_27, FUS_34 and FUS_40 families SNP variance consistent with the donor parent and recurrent parent between UMN10 and gwm493 in resistant and check families respectively. Only in FUS_12 families, resistant and check, were there observed discrepancies between those two marker systems.

### 2.6. Association Weight Matrix Results

**Figure 6:**
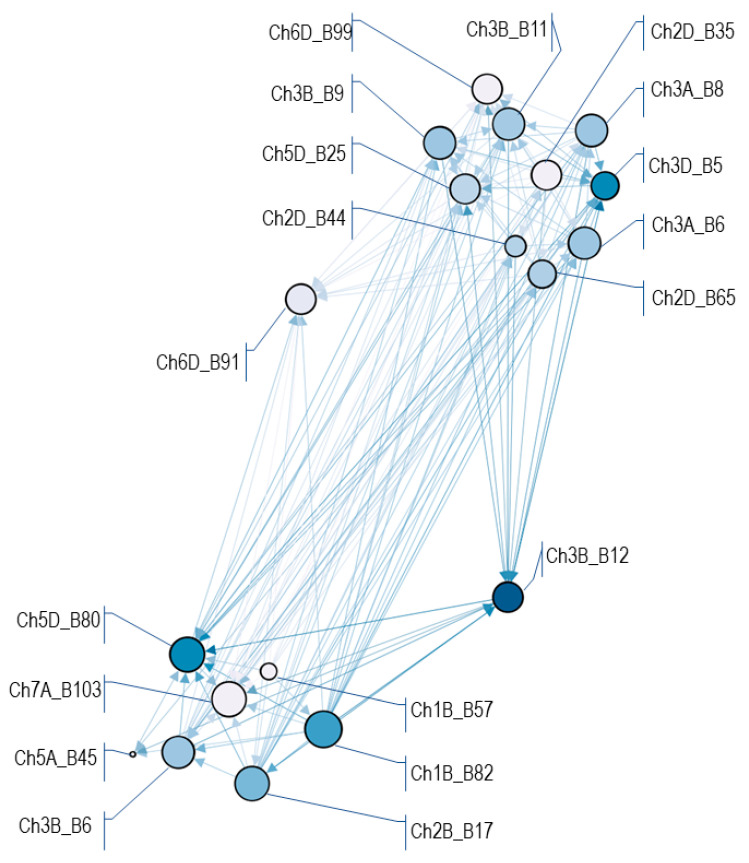
Network prepared from GWAS results obtained from FHB resistance data and DArTseq genotyping of wheat family panel using the association weight matrix and partial correlation–information theory approaches. Nodes represent 19 haploblocks and edges represent significant correlations between HBs. Colors of the nodes indicate influential nodes for the highest value.

After filtering, the 10 phenotypes and 19 HBs (for detailed information see Table S3) were used for association weight matrix creation. The OpenOrd filtering followed by the PCIT algorithm allowed us to obtain 279 edges connecting to 19 nodes (Figure 6) with various influence (from 0 to 5.9 of betweenness centrality metrics) and numbers of connections from 4 to 18. Nodes were structured into two pools and two outliers. Two nodes, Ch2D_B35 and Ch3B_B9, represented HTs identified as significant in GWAS, Ch2D_B35_H2 and Ch3B_B9_H2, which increased the phenotypic value (increased susceptibility). The nodes Ch2D_B35 and Ch3B_B9 were clustered within one pool with each other and the other eight HBs.

## 3. Discussion

The search for varieties resistant to Fusarium head blight is crucial for wheat breeders all over the world, as wheat is one of the most important crops. Breeding strategies based on molecular selection enable the most effective solutions. As the number of mapped QTL increases, MAS become an attractive and successful solution and the application of MAS for improving FHB resistance has been reported e.g., [23,24,25,26]. The use of an FHB resistance donor enables resistance to be introduced into new materials. One of the first sources of FHB resistance was the Asian variety Sumai3, in which QTL *Fhb1* (*Qfhs.ndsu-3BS*), with the main effect determining type II resistance, was identified [37,38]. The phenotypic results of the present research, in comparison to check families (without the *Fhb1* gene), show a significant influence of type II resistance in F_3_BC_2_ (2019) and F_4_BC_2_ (2020) families selected for having the resistance gene *Fhb1* (Figure 1a). The higher plant resistance may also be correlated with lower deoxynivalenol (DON) production in plants enriched with the *Fhb1* locus, as those plants detoxify DON by DON-glycoside production [9,39,40]. We noted similar results in the case of mixed resistance of type I and type II (Figure 1b), which shows that type II resistance had a major influence on the field resistance. The resistant wheat lines and cultivars used by the authors as resistant checks showed reactions at a similar level to investigated Fhb1 wheat families. This indicates accurate molecular selection of plant material possessing the *Fhb1* resistance gene. Comparing the five combinations of examined wheat families, FUS_12 showed the significantly lowest level of *F. culmorum* infection. This creates the opportunity for utilization of the resultant data in MAS.

Many researchers have investigated interactions between chromosome regions and their influence on plant response to Fusarium head blight [24,34,41]. Marker-assisted backcrossing has been used to improve FHB resistance. Salameh et al. [24] studied the influence on infestation and interactions of two best known QTL, *Fhb1* and *Qfhs.ifa-5A*, in winter wheat lines. They observed that interaction between these crucial QTL is significant for the response and showed a positive additive effect. Li et al. [41] identified one major QTL on 7D on four minor QTL with positive additive effects. Arruda et al. [34] defined effects of favorable alleles associated with phenotypic traits on seven chromosomes ranging from 1.36 to 9.54. In our study, there were haplotypes associated with different traits indicating a positive or negative effect (Table 3). Deeper insight into these details allows specific genotypes to be defined.

As an alternative to QTL mapping, a genome-wide association study (GWAS) can be applied. However, the GWAS has low power to detect rare alleles, even those with a large phenotypic effect [42]. GWAS based on linkage disequilibrium (LD) has been widely used to discover complex agronomic traits. The innovative approach to association studies based on haploblocks and haplotypes has so far been rarely published. There are few works available that especially address Fusarium head blight resistance. Haplotype-GWAS has been used in identifying genes controlling complex traits. Useful genetic regions associated with investigated traits could be a powerful tool for MAS breeding. There has been an emphasis on haplotype association studies in many crops, including rice [43], barley [44], maize [45] and wheat [21,46]; this promises to greatly accelerate crop improvement if properly deployed.

Advanced analysis in our work revealed significant associations of molecular markers and phenotypic traits related to resistance to Fusarium head blight. The most informative for our approach was type II resistance across two years of research. Moreover, other traits mentioned earlier were taken into account. Four crucial associations were determined on three chromosomes. The emerged haplotypes had various effects on the traits associated with FHB resistance (Table 3). Chromosome 3BS is the most interesting for us, being the one where the *Fhb1* gene is located. The main goal of this study was incorporation of the mentioned gene into Polish wheat breeding lines, so the resultant association is crucial for the breeding approach. The *Fhb1* locus is located within the region selected using microsatellite markers during the first step of analysis. The SSR variants were consistent with DArT-SNP markers at a satisfactory level in four of the five FUS families. The MTA detected on 3BS was connected with type II resistance and it was at the distance of about 5 Mb from the resistance gene *Fhb1*, according to the location of the central marker UMN10 (8,522,947–8,522,966 bp) in EnsemblPlants [47]. The lack of HB coverage of the *Fhb1* locus may be due to lower DArT-SNP marker coverage in this region (Figure 4); nevertheless, the distance between UMN10 and the nearest HB is relatively short. Haplotype Ch3B_B9_H2 (13,276,831–13,633,016 bp) described in this work was within the part of the short arm of chromosome 3B selected by SSR markers. The presence of Ch3B_B9_H2 significantly increased the values of Type_2_2020 (Figure S4c) and Type_2_2019&2020 (Figure S5c) traits. This result is not surprising, as the HT was represented only in check families lacking *Fhb1* (Table S2). Arruda et al. [34], as a result of GWAS based on LD, reported a highly significant marker on 3BS associated with resistance at the *Fhb1* locus. The authors also selected haplotypes presenting favorable or unfavorable effects of alleles for different traits: SEV, INC and DON (severity, incidence and deoxynivalenol concentration) associated with Fusarium head blight resistance. Similarly to Arruda et al. [34], Li et al. [48] reported that most of the loci identified during association mapping coincided with previously published QTL, not only on 3BS but also on chromosome 2D. Haplotype analysis of the 3BS genomic region involved in FHB resistance was applied by Hao et al. [21]. The results of their study showed that the *Fhb1* resistance gene is located within the described haploblock. They suggest that strong interaction between two haplotypes (HapB3-2 and HapB6-2) leads to increased FHB resistance. Our results indicate close location of *Fhb1* and Ch3B_B9_H2, giving the potential for selected wheat families and providing useful information for MAS in wheat breeding programs.

Other significant associations have been identified on chromosome 2DS. In the current study we identified two haplotypes—Ch2D_B35_H1 and Ch2D_B35_H2—in haploblock Ch2D_B35 (32,388,539–32,388,595 bp) (Figure 5d) associated with FHB resistance traits, mainly resistance type II. Ch2D_B35_H1 has a positive effect in the represented families (Table 3) with the phenotypic variance ranging from 41 to 43% (Table 2). On the other hand, haplotype Ch2D_B35_H2 shows the opposite effect (Figure 5c). This is the expected reaction, as it is represented in check families (Table S2). Li et al. [48] revealed the associations on 2DS as a result of GWAS analysis, and they were concerned with the evaluation of type II FHB resistance. The investigated marker (Xgdm35, 13,754,175–13,754,194 bp) was reported to be associated with FHB resistance QTL and was shown to be responsible for the plant susceptibility. However, in this work, only marker–trait associations were considered (not haploblocks), and the genetic distance compared to our results is high. Considering other recognized chromosomal regions with regard to resistance to FHB, to date *QFhs.nau-2DL* (470,232,616–577,440,823 bp) with a high phenotypic effect has been mapped on the long arm of chromosome 2D [49]. Jiang et al. [49] researched PPS (percentage of scabby spikelets) and NSS (number of scabby spikelets) as a measurements of type II resistance to FHB. They detected a positive additive effect of a 2DL QTL (*QFhs.nau-2DL*) as well as a 3BS QTL. Somers et al. [50] also previously identified the QTL *QFhs.crc-2DL* on 2DL controlling FHB symptoms and DON accumulation; whether it is the same QTL as *QFhs.nau-2DL* is unclear [51]. Balut et al. [52] reported that a QTL on 2DL (561,157,470–561,157,752 bp) significantly reduced FDK (*Fusarium* damaged kernels) in investigated populations and was associated with DON reduction, indicating its positive effect on FHB resistance. The physical position of this mentioned QTL is greatly different from the position of HB on 2DS significantly associated in our experiment. However, it is crucial to consider that the location of HBs described in our work was physical, whereas others report the length of the genetic (cM) regions. The comparison was possible on the basis of the physical locations of the selected markers in the EnsemblPlants database [47].

The last marker–trait association was described on chromosome 7DL. The haplotype Ch7D_B63_H4 (439,405,257–444,190,466 bp) was associated with type II resistance (Type_2_2020) and explained 34% of the phenotypic variance (Table 2). Its negative effect on the trait was obvious when considering that only check families represent the haplotype (Table S2). So far, very few associations or even QTL for FHB resistance have been identified on chromosome 7D. A GWAS resulting in associations on 7DS was published by Arruda et al. [34]. They identified an SNP significantly associated with disease incidence, accounting for 16% of the variance, and lines carrying multiple favorable alleles showed lower levels of disease. Eckard et al. [53] applied the IBD (identity-by-descent) linkage analysis method, as well as haplotype analysis for each chromosome for a genome-wide QTL scan. Among the identified QTL was *Qfhb.sdsu-7D* (7DS), with a minor effect explaining 3.4–5.3% of phenotypic variance of FHB resistance traits. A Chinese spring wheat landrace with superior resistance to FHB was used by Li et al. [41] in crosses as a resistance source. The wheat progeny was evaluated for FHB incidence. As a result of composite interval mapping, a major QTL on 7D was identified, *QFhb.hyz-7D*, contributing resistance to FHB (lower percentage of scabbed spikelets). This major 7DS showed an additive effect, but its location (132,197,517–132,197,538 bp) significantly differs from the HB association position described in our work.

A genetic network (Figure 6) was generated in order to present the genetic relationship between haploblocks and their influence on phenotypic traits to exploit the results of GWAS. In comparison to single trait GWAS, this analysis revealed more opportunities and it also identified relevant genes that would have been missed by single-trait GWAS. The AWM provided a prediction of gene interactions based on HB effect correlations. Short edges display positive correlation within nodes, whereas a strong negative correlation is displayed as a large distance between them. In our study, the quantitative character of *Fhb1* resistance depends on a complex network formed by at least 19 loci. The HB Ch3B_B9, the closest to *Fhb1*, is clustered with Ch2D_B35 and eight other HBs. As long as those two nodes represented HTs with a negative phenotypic effect, there is evidence that all of the collocated HBs participated in wheat susceptibility, opposite to the second seven-node cluster. Nevertheless, the AWM was prepared for haploblocks while the GWAS referred to haplotypes, therefore deeper insight is needed for identification of HB interactions.

## 4. Materials and Methods

### 4.1. Plant Material

In the study, as a source of the *Fhb1* resistance gene, winter wheat line AIII62 (F_4_BC_2_ generation) was used, herein designated as P_FUS_9. This line was obtained by the MABC approach from a cross between Chinese spring wheat Sumai3 and the Polish winter wheat cultivar Muszelka where presence of the *Fhb1* locus was controlled by the closely linked marker UMN10 developed by Liu et al. [19] (unpublished data).

**Figure 7:**
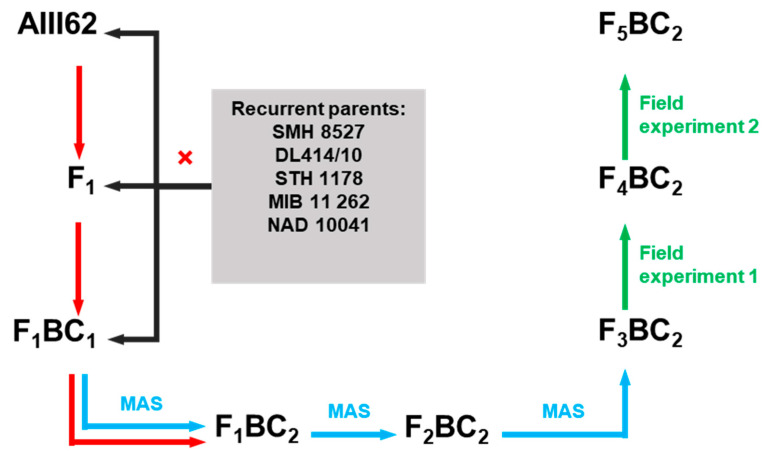
Scheme for marker-assisted backcross selection to introduce *Fhb1* resistance gene into winter wheat. Red arrows indicate crosses, blue arrows indicate MAS (marker-assisted selection), green arrows indicate field experiments.

Polymorphism in the central and flanking marker loci on chromosome 3B between the donor line and 34 recurrent parents (RPs) allowed us to choose the five best winter wheat breeding lines originating from each of the five Polish plant breeding companies (Table S4) that were used in further crosses. We included all five breeding companies also to provide the opportunity to represent presumably the widest possible Polish winter wheat gene pool. Consecutive crosses and backcrosses were made in the years 2014–2018 (Figure 7). The F_1_ generation was backcrossed with RP in each combination. After molecular selection, generation F_1_BC_1_ was again crossed with RP to obtain F_1_BC_2_. Next, F_2_BC_2_ and F_3_BC_2_ were generated, but at each step, molecular selection was performed in order to select lines with the desired allele profile. During 2019–2020, field tests were conducted on F_3_BC_2_ and F_4_BC_2_ generations (Figure 7).

Generations F_3_BC_2_ (2019) and F_4_BC_2_ (2020) of each of the five combinations as described—FUS_12 (SMH 8527) with 2 families, FUS_24 (DL 414/10) with 6 families, FUS_27 (STH 1178) with 16 families, FUS_34 (MIB 11 262) with 13 families and FUS_40 (NAD 10041) with 12 families—were tested for FHB resistance with two isolates. Additionally, 3 families from each combination (F_3_BC_2_ in 2019 and F_4_BC_2_ in 2020) without *Fhb1* (15 check families) were also phenotyped.

In order to determine the general degree of disease severity, several additional groups of wheat (lines and cultivars) were added to the experiment:

- Winter wheat cultivars: Belenus (France), Belissa (Poland), Błyskawica (Poland), Ceres—durum (Poland), Euforia (Poland), Opcja (Poland), Plejada (Poland), Reduta (Poland), Sfera (Poland), Tobak (Germany), Wilejka (Poland) [54];
- Wheat resistant checks, wheat lines carrying the *Fhb1* gene: UNG 136.6.1.1 [Fhb1+] (Hungary), S 10 [Fhb1+] (Poland), S 30 [Fhb1+] (Poland), S 32 [Fhb1+] (Poland) and wheat breeding lines without the *Fhb1* gene: 20,828 [Fhb1-] (Austria) and A40-19-1-2 (Austria) and cultivars Arina (Switzerland) and Fregata (Poland) [54];
- Wheat susceptible checks, Polish breeding lines: SMH 8694 (S) (Poland), SMH 8816 (S) (Poland), DL325/11/3 (S) (Poland), KBP 14 16 (S) (Poland) [55].

### 4.2. Fungal Material and Field Experiments

Inoculum production has been described by Góral et al. [55]. Isolates were increased on autoclaved wheat grain in glass Erlenmeyer flasks for one week at 20 °C in darkness and then exposed to near UV light under a 16 h photoperiod for three weeks at 15 °C.

The inoculum consisted of 2 isolates of *F. culmorum* 3ADON chemotype: KF 846 (deoxynivalenol (DON) chemotype) originating from the collection of the Institute of Plant Genetics, Polish Academy of Science (Poznań, Poland) and ZFR 112 (DON chemotype, producing high amount of zearalenone (ZEN) in vitro) originating from the collection of Plant Breeding and Acclimatization Institute—National Research Institute (PBAI-NRI) (Radzików, Poland). These isolates have proven their aggressiveness against wheat under field conditions on several occasions [56,57,58,59].

Two-year experiments were conducted under polytunnel conditions in an experimental field at Radzików (PBAI-NRI). The 49 selected F_3_BC_2_ (2019) and F_4_BC_2_ (2020) derived wheat families and 15 check wheat families (the same generations) were inoculated. In addition, wheat cultivars and breeding lines, as well as susceptible and resistant checks were tested as a reference (described above) for FHB infection level.

To determine type I and type II FHB resistance, the selected lines were sown in two experiments under partially controlled conditions in polytunnels with a mist irrigation system. Lines were sown in 1-row plots 1 m long without replications. The spacing between rows was 30 cm. For type I resistance wheat at the full flowering phase, the heads were sprayed with a conidial suspension of two mixed *F. culmorum* isolates adjusted to 10^5^ spores/mL. The number of infection points on 10 ears per plot was assessed 7 to 10 days after inoculation [60]. To evaluate type II resistance, the heads were inoculated at the full flowering phase by placing a drop (approx. 50 µL) of a suspension of *F. culmorum* spores (concentration 50 × 10^3^ spores/mL) in the flower of the middle spikelet of the labelled heads [61]. Each isolate was inoculated with 5 ears of a given line. The severity of spike infestation was assessed by determining the number of spikelets with symptoms of the disease. Assessment was carried out 21 days after inoculation. During the experiment, high air humidity stimulating the development of the disease was maintained by means of an irrigation system (applied after inoculation). Three weeks after inoculation on 20 randomly selected heads from each plot, disease progress was visually evaluated as the Fusarium head blight index (FHBi) [55]:

$$\mathrm{FHBi}=\frac{\%\;\mathrm{of}\;\mathrm{head}\;\mathrm{infection}\;x\;\%\;\mathrm{of}\;\mathrm{heads}\;\mathrm{infected}\;\mathrm{per}\;\mathrm{plot}}{100}$$

In total, ten phenotypic traits were evaluated: Type_1_2019 for type I resistance in 2019, Type_2_2019 for type II resistance in 2019, Type_1&2_2019 for mixed type I and type II resistance in 2019, Type_1_2020 for type I resistance in 2020, Type_2_2020 for type II resistance in 2020, Type_1&2_2020 for mixed type I and type II resistance in 2020, Type_1_2019&2020 for type I resistance in 2019 and 2020, Type_2_2019&2020 for type II resistance in 2019 and 2020, Type_1&2_2019&2020 for type I and type II resistance in 2019 and 2020. Due to unfavorable weather conditions, FHBi data were only available for the year 2019.

### 4.3. Statistical Analysis

The statistical analysis was performed using XLSTAT Life Science, Version 2021.2.1.1119 (Addinsoft, New York, NY, USA). Normality of the phenotypic data distribution was tested using the Shapiro–Wilk test (XLSTAT procedure: Normality test). None of the variables followed a normal distribution and they were log10 transformed (type I, type II, type I and II). Additionally, data for FHBi were Box–Cox transformed.

Type I resistance, type II, type I and II and FHBi data were analyzed by means of analysis of variance (ANOVA) using XLSTAT to distinguish analyzed groups and visualize statistically significant differences. Tukey’s honestly significant difference (HSD) test was used for analysis of the differences between groups with a confidence interval of 95%.

Box plots enable visualization of phenotypic differences between analyzed groups and were drawn on raw data. Comparison of the means of different wheat groups was done using Tukey’s HSD test. Statistical significance is indicated with letters; boxes marked with the same letter are not significantly different at *p* < 0.05 according to Tukey’s test performed on log10 or Box–Cox transformation.

### 4.4. Genetic Marker Selection

Well-characterized molecular markers associated with the part of chromosome 3BS linked to *Fhb1*, namely the central marker UMN10 and flanking SSR markers—distally located gwm389 and proximally located barc12, gwm493 and gpw3248—were used in the MABC strategy. At each step of molecular selection (F_1_BC_1_—600 plants, F_1_BC_2_—600 plants, F_2_BC_2_—600 plants), plant DNA was extracted using a DNeasy Plant Mini Kit (QIAGEN GmbH, 140724 Hilden, Germany), Nucleo Mag 96 (Marcherey-Nagel GmbH & Co. KG, 52355 Düren, Germany) with changes [62] using a Freedom Evo robotic workstation (Tecan Group Ltd., Seestrasse 103, CH 8708 Männedorf, Switzerland) or CTAB [63]. Molecular analysis was divided into two steps. Firstly, plants were tested for the central marker UMN10 and in F_1_BC_1_ and F_1_BC_2_ generations heterozygous lines in this locus were selected and for F_2_BC_2_ homozygous lines (in the type of the gene donor parent). Secondly, these lines were tested with flanking markers, distally located gwm389 and at least one from the proximally located barc12, gwm493 and gpw3248, and lines homozygous in the tested SSR loci in the type of RP were chosen. The PCR reaction conditions were described by Czembor et al. [62]. Some of the molecular markers were amplified with the M13 system according to Rampling et al. [64]. The PCR products were separated and detected on 4.75% denaturing polyacrylamide gels (Long Ranger, Cambrex Bio Science, USA) using an ABI Prism 377 DNA sequencer (Applied Biosystems, Foster City, CA, USA).

The last step of this work was detailed molecular analysis of the selected genotypes from the F_3_BC_2_ generation using DArTseq technology (Diversity Arrays Technology P/L, Bruce, Australia) [65]. Eight parental lines, i.e., SMH 8527 (P_FUS_12), DL 414/10 (P_FUS_24), STH 1178 (P_FUS_27), MIB 11 262 (P_FUS_34), NAD 10041 (P_FUS_40) and AIII62 (P_FUS_9), its parental genotypes Muszelka and Sumai3 and the next 35 genotypes of F_3_BC_2_ with the desired arrangements of alleles (central marker UMN10 in the type of gene donor parent and flanking markers in the type of RP respectively for each combination) were analyzed. Additionally, three control objects (check families) for each combination were included (15 objects), genotypes from the F_3_BC_2_ generation that, after SSR selection, possessed only alleles from the recurrent parent (lacking the *Fhb1* gene). In total, 58 DNA samples were subjected to DArTseq analysis.

### 4.5. Haploblock Construction and GWAS Analysis

DArT-SNP markers with known genomic location (DArT Wheat_ChineseSpring20 reference model provided by Diversity Arrays Technology P/L) and with missing data below 20% were used. In the next step, the imputation was conducted with A.mat R function from the rrBLUP R package [66] using an expectation maximization (EM) algorithm based on the multivariate normal distribution while removing markers with minor allele frequency (MAF) < 5%. Afterwards, based on the solid spine of linkage disequilibrium (LD) and extended spine if D’ > 0.8, further analysis was prepared with haploblocks (HB) designated with Haploview 4.2 [67]. Next the Haploview resultant data were converted into the 0/1 matrix format by Haploview2gapit Python script [68]. The haplotypes with MAF < 5% were discarded from further analysis. Finally, the visualization was prepared in Haploview 4.2 [67] and Flapjack 1.21.02.04 software [69].

Principle component analysis (PCA) and kinship similarity matrix preparation were conducted with PCA and K.mat R functions respectively from the rrBLUP R package [66]. The GWAS analysis was prepared using the first five PCs with the GWAS function from the rrBLUP R package [66] employing a similarity matrix. GWAS analysis was performed on 10 phenotypic traits (Type_1_2019, Type_2_2019, Type_1&2_2019, Type_1_2020, Type_2_2020, Type_1&2_2020, Type_1_2019&2020, Type_2_2019&2020, Type_1&2_2019&2020 and FHBi). Marker–trait associations were calculated separately for each of them. The Manhattan and Q–Q (quantile–quantile) plots were visualized in the qqman R package [70]. The Bonferroni correction (-log(*p*) > 5.22) was used as a threshold for significant HB trait associations. The explanation of phenotypic variance (R^2^) was calculated for significant HB in the CJAMP R package [71].

### 4.6. Association Weight Matrix Pipeline

To exploit the GWAS obtained data, the association weight matrix (AWM) pipeline [72] was employed. Type2_2019&2020 was selected as a key phenotype and one haplotype (HT) was selected for representing one haploblock (HB) according to the lowest p-value for the highest number of associated phenotypes. For the analysis, the HBs associated with the key phenotype and at least 50% of traits were selected. The distances from the genes of all the HBs were considered as 0 kb. The partial correlation–information theory (PCIT) algorithm [73] was employed to calculate interactions between HBs. The visualization of the network was prepared in Gephi 0.9.2 [74] according to the OpenOrd protocol [75] with the following parameters: edge cut = 0.95, number of iterations = 850 and filtering for sparse correlations values ≥ 0.80. The color of the nodes indicates influential nodes for the highest value according to betweenness centrality metrics. The size of the nodes represents degree centrality, which is the number of connections.

## 5. Conclusions

Incorporation of the *Fhb1* gene into wheat breeding lines was successful. All the propagated families which were suspected to carry *Fhb1* according to SSR variants revealed higher resistance to FHB than susceptible checks. Despite the high-density genotyping techniques available, the usage of well-known SSR markers is effective. The haplotype-based GWAS allowed us to identify loci associated with Fusarium head blight resistance. The high level of explained phenotypic variance by identified MTA makes them promising and interesting for breeders. While the GWAS analysis allowed us to reveal loci associated with, at most, three resistance traits in our study, the AWM employed all analyzed phenotypes during one pipeline’s run. The genetic network of significant haploblocks showed the complexity of the wheat response to Fusarium head blight, which requires further investigations and caution during the breeding process. The results obtained in this work can be a prelude to the search for regions potentially affecting wheat resistance. The use of haploblocks in the GWAS method is more advantageous and leads to better, more accurate results comparing to SNP based GWAS. These novel molecular solutions give more perspective, and new possibilities for scientists and breeders that could contribute to further effective management in disease control.

## Figures and Tables

**Table 1:** Detailed information about the haploblock-based map for 58 genotypes of wheat based on DArT markers.

Chromosome	SNP ^1^	HB ^2^	SNPs per HB avg.	SNPs per HB max	HT ^3^	HB Size avg. (Mb)	HB Size max. (Mb)	Chromosome Coverage (%)
Chr1A	340	82	3.5	12	288	3.1	36.2	42.7
Chr1B	424	96	3.8	16	363	4.1	54.9	57.7
Chr1D	331	85	3.3	12	330	3.8	62.7	64.8
Chr2A	593	115	4.6	26	318	3.1	28.6	45.4
Chr2B	717	126	5.2	58	444	3.6	30.6	57.1
Chr2D	581	139	3.5	19	513	1.8	26.1	38.9
Chr3A	451	89	4.3	26	346	3.8	26.2	45.5
Chr3B	633	138	3.8	13	477	3.5	49.2	57.3
Chr3D	475	104	3.4	25	403	2.3	20.3	38.6
Chr4A	340	82	3.4	19	306	4.9	32.6	54.1
Chr4B	338	72	3.7	22	261	5.0	102.9	53.9
Chr4D	223	46	3.1	9	199	3.3	40.8	29.6
Chr5A	529	123	3.7	30	444	3.2	115.1	55.7
Chr5B	583	117	4.3	50	412	3.4	33.2	56.6
Chr5D	409	88	3.8	13	354	3.3	27.1	51.6
Chr6A	373	74	4.2	25	284	3.5	25.4	42.0
Chr6B	580	131	3.5	16	480	3.2	26.2	57.7
Chr6D	404	101	3.2	9	395	2.2	27.9	46.8
Chr7A	667	141	4.0	19	543	2.6	45.9	50.2
Chr7B	513	123	3.4	20	477	3.0	31.7	48.7
Chr7D	516	120	3.3	13	476	2.1	18.3	38.7
ChrUn ^4^	231	64	2.8	8	260	2.0	10.9	26.5

^1^ single nucleotide polymorphism; ^2^ haploblock; ^3^ haplotype; ^4^ chromosome unknown.

**Table 2:** GWAS results for 58 wheat lines indicating haplotypes associated with Fusarium head blight resistance parameters.

No	Chromosome	HT *	Locus (bp)	*p*-Value	Trait	R^2^
1	2D	Ch2D_B35_H1	Chr2D:32388539-32388595	6.98948 × 10^−7^	Type_2_2020	41%
2				5.56168 × 10^−7^	Type_2_2019&2020	43%
3				3.8519 × 10^−6^	FHBi	6%
4	2D	Ch2D_B35_H2	Chr2D:32388539-32388595	2.8397 × 10^−7^	Type_2_2020	41%
5	2D			3.8328 × 10^−6^	Type_1&2_2020	39%
6	2D			1.7512 × 10^−7^	Type_2_2019&2020	46%
7	3B	Ch3B_B9_H2	Chr3B:13276831-13633016	1.9527 × 10^−7^	Type_2_2020	46%
8	3B			2.22 × 10^−7^	Type_2_2019&2020	49%
9	7D	Ch7D_B63_H4	Chr7D:439405257-444190466	1.6867 × 10^−6^	Type_2_2020	34%

* haplotype.

**Table 3:** Haplotypes described by GWAS and their effects on FHB related traits.

Trait	Haplotype	Chromosome	Freq. Effect ^1^
Type_2_2020	Ch2D_B35_H1	2D	33 +
	Ch2D_B35_H2	2D	21 −
	Ch3B_B9_H2	3B	21 −
	Ch7D_B63_H4	7D	5 −
Type_2_2019&2020	Ch2D_B35_H1	2D	33 +
	Ch2D_B35_H2	2D	21 −
	Ch3B_B9_H2	3B	21 −
Type_1&2_2020	Ch2D_B35_H2	2D	21 −
FHBi	Ch2D_B35_H1	2D	33 +

^1^ Frequency effect refers to the number of genotypes that possess an identified haplotype; (+) indicates that the haplotype reduces the phenotypic value (increasing the resistance); effect (−) indicates that the haplotype increases phenotypic value (increasing susceptibility).

## Data Availability

Data sets analyzed during the current study are available from the correspondent author on reasonable request.

## Supplementary Materials

The following supporting information can be downloaded at: https://www.mdpi.com/article/10.3390/ijms232214233/s1.
