# Supplementary material for: Evaluation of Fusarium Head Blight Resistance Effects by Haplotype-Based Genome-Wide Association Study in Winter Wheat Lines Derived by Marker Backcrossing Approach
Source: Int J Mol Sci. 2022 Nov 17;23(22):14233. doi: 10.3390/ijms232214233 (PMC9695032; doi:10.3390/ijms232214233)
Supplement: Supplementary file 1 [file ijms-23-14233-s001.zip › Suppl Figures.pdf]

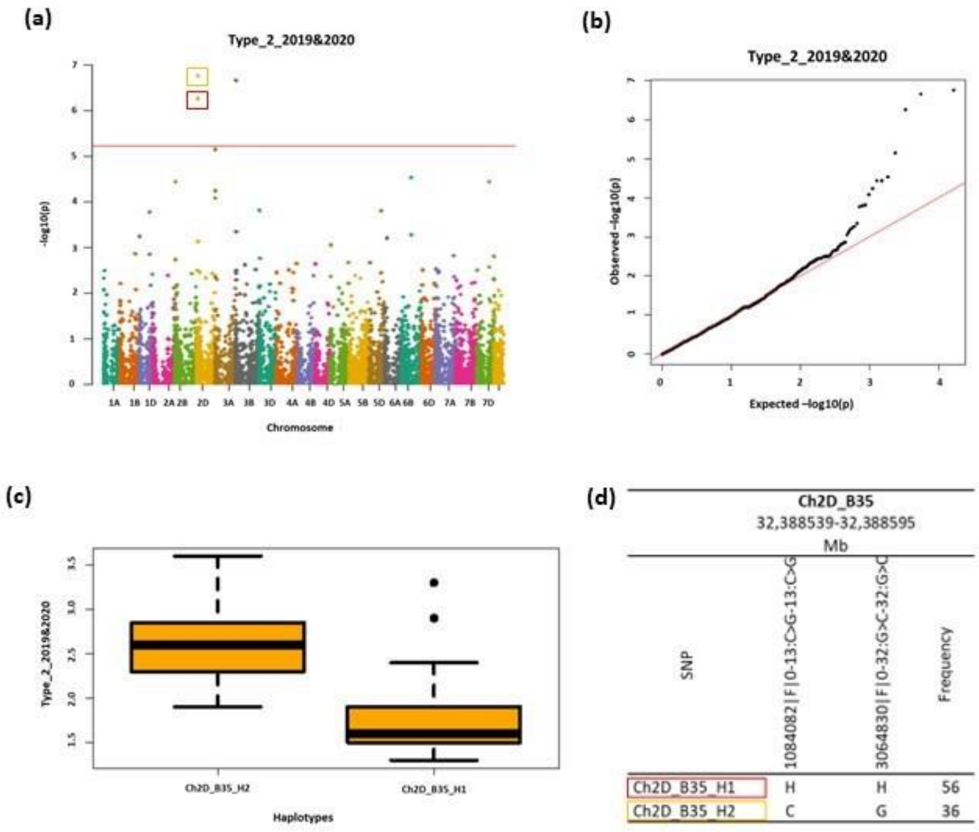

Figure S1. (**a**) Manhattan plot on 21 wheat chromosomes for Type_2_2019&2020 trait. Red horizontal dash represents the GWAS threshold of -log(p) = 5.22 for Bonferroni correction and dots above the red line indicate significant MTAs. (**b**) Q-Q plot for GWAS results for Type_2_2019&2020 trait. (**c**) Boxplots indicating the phenotype value of Type_2_2019&2020 trait corresponding to the two haplotypes (H1 and H2) in the same haploblock (Ch2D_B35) on chromosome 2D. (**d**) Haplotype block based on two SNP markers on chromosome 2D; both haplotypes are significantly associated with the Type_2_2019&2020 trait (MTA). Frequency shows occurrence (percentage) of haplotypes in the group of families.

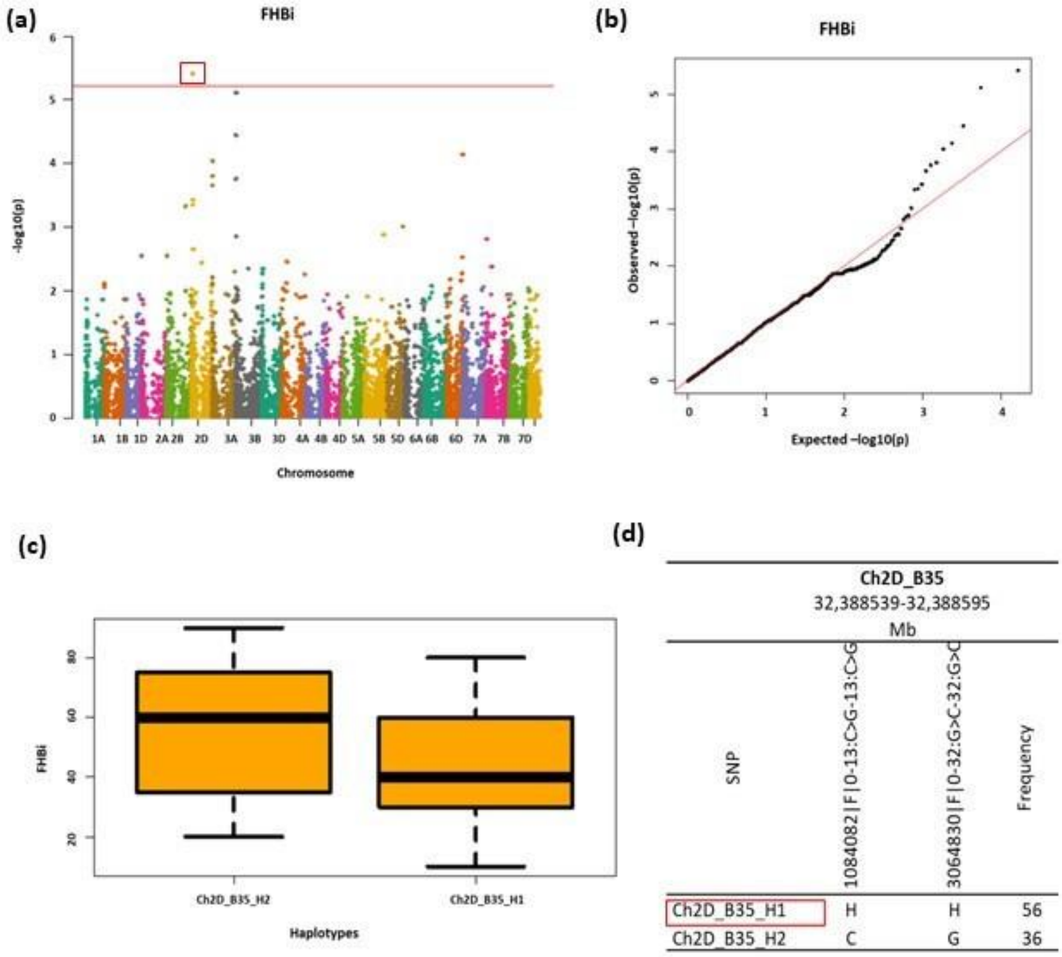

Figure S2. (**a**) Manhattan plot on 21 wheat chromosomes for FHBi trait. Red horizontal dash represents the GWAS threshold of -log(p) = 5.22 for Bonferroni correction and dot above the red line indicate significant MTA. (**b**) Q-Q plot for GWAS results for FHBi trait. (**c**) Boxplots indicating the phenotype value of FHBi trait corresponding to haplotypes in the haploblock Ch2D_B35 on chromosome 2D. (**d**) Haplotype block based on two SNP markers on chromosome 2D; haplotype significantly associated with the FHBi trait (MTA) marked with red. Frequency shows occurrence (percentage) of haplotypes in the group of families.

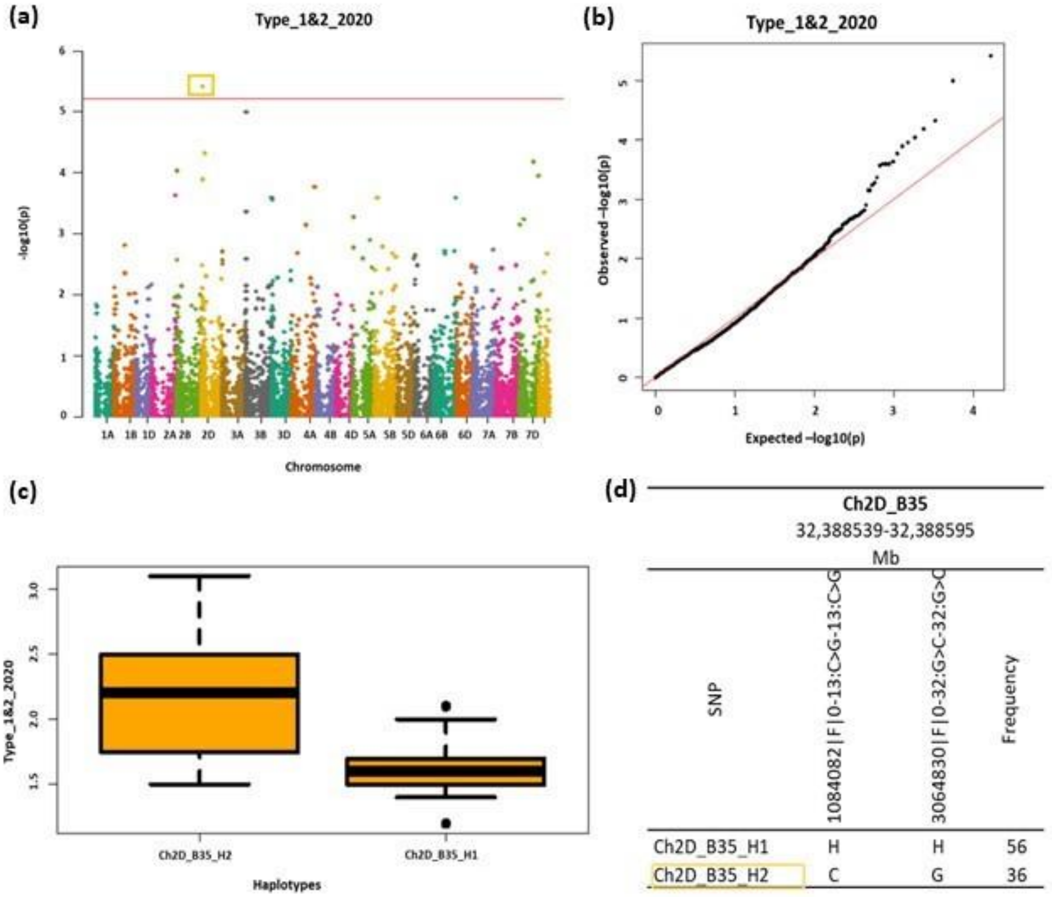

Figure S3. (**a**) Manhattan plot on 21 wheat chromosomes for Type_1&2_2020 trait. Red horizontal dash represents the GWAS threshold of -log(p) = 5.22 for Bonferroni correction and a dot above the red line indicate significant MTA. (**b**) Q-Q plot for GWAS results for Type_1&2_2020 trait. (**c**) Boxplots indicating the phenotype value of Type_1&2_2020 trait corresponding to haplotypes in the haploblock Ch2D_B35 on chromosome 2D. (**d**) Haplotype block based on two SNP markers on chromosome 2D; haplotype significantly associated with the Type_1&2_2020 trait (MTA) marked with yellow. Frequency shows occurrence (percentage) of haplotypes in the group of families.

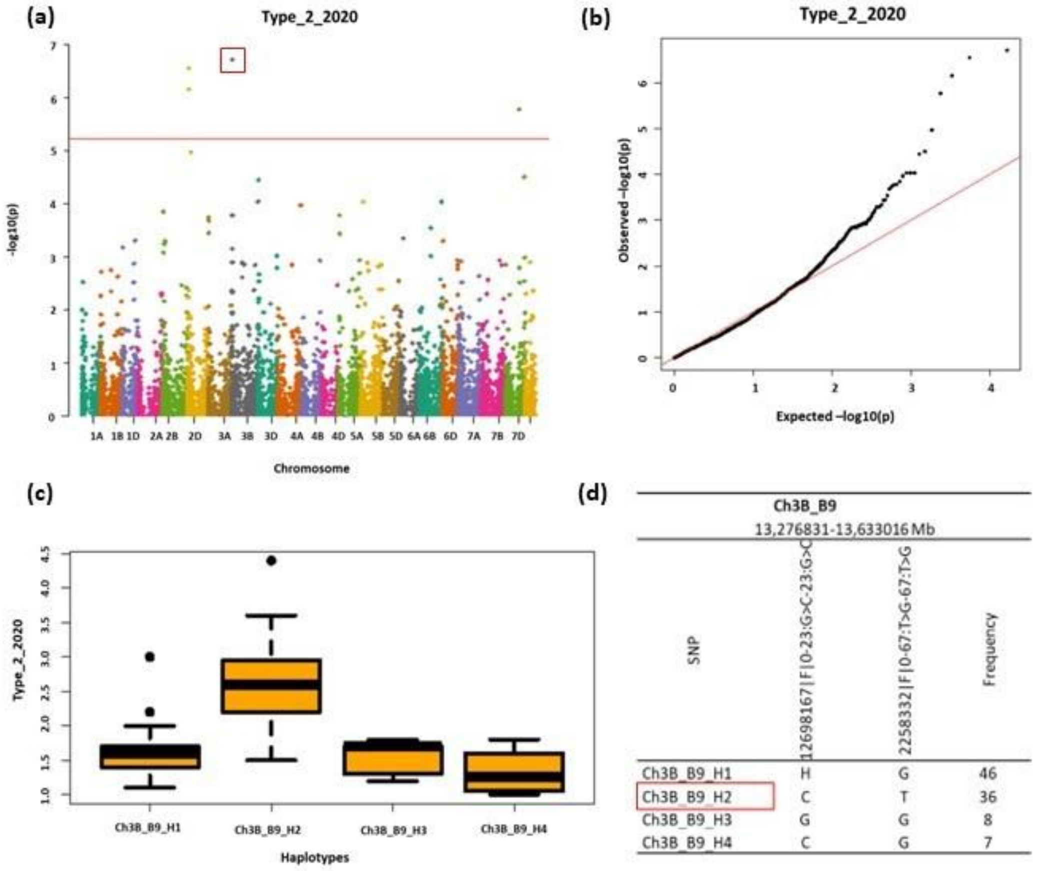

Figure S4. (**a**) Manhattan plot on 21 wheat chromosomes for Type_2_2020 trait. Red horizontal dash represents the GWAS threshold of -log(p) = 5.22 for Bonferroni correction and dots above the red line indicate significant MTAs. (**b**) Q-Q plot for GWAS results for Type_2_2020 trait. (**c**) Boxplots indicating the phenotype value of Type_2_2020 trait corresponding to haplotypes in the haploblock Ch3B_B9 on chromosome 3B. (**d**) Haplotype block based on two SNP markers on chromosome 3B; haplotype significantly associated with the Type_2_2020 trait (MTA) marked with red. Frequency shows occurrence (percentage) of haplotypes in the group of families.

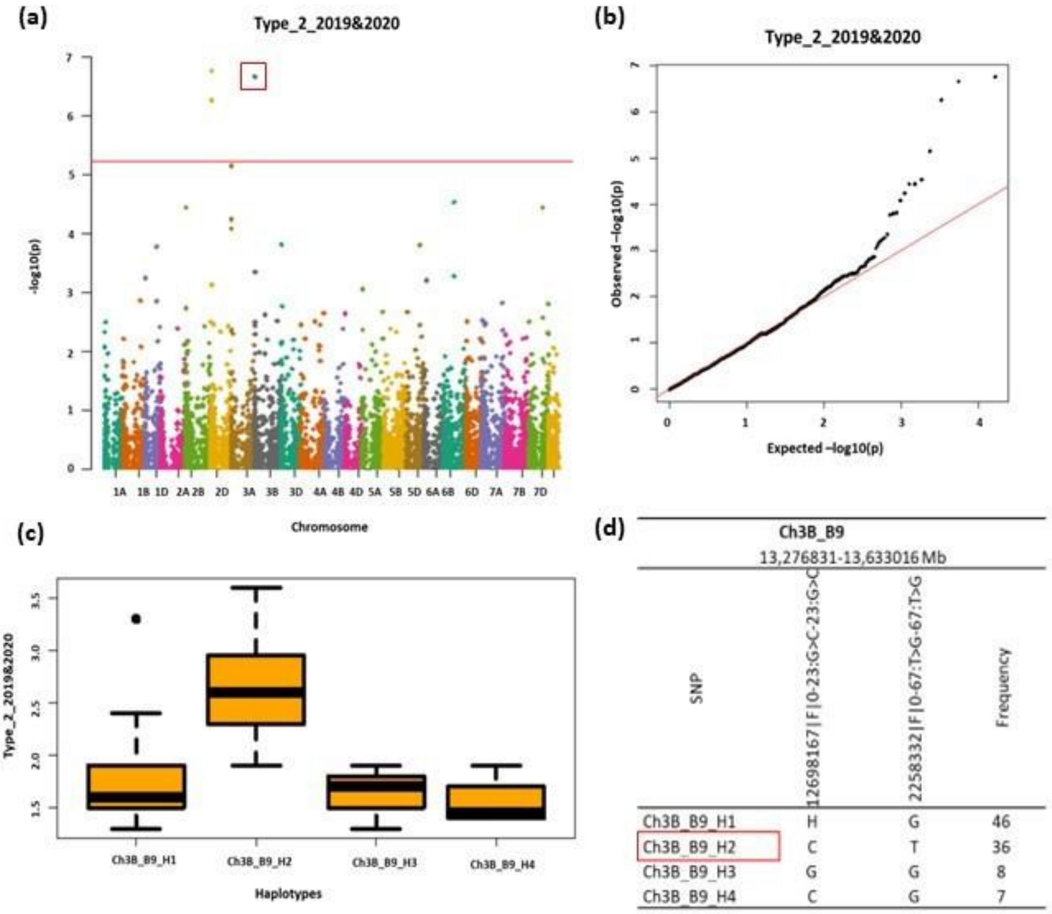

Figure S5. (**a**) Manhattan plot on 21 wheat chromosomes for Type_2_2019&2020 trait. Red horizontal dash represents the GWAS threshold of -log(*p*) = 5.22 for Bonferroni correction and dots above the red line indicate significant MTAs. (**b**) Q-Q plot for GWAS results for Type_2_2019&2020 trait. (**c**) Boxplots indicating the phenotype value of Type_2_2019&2020 trait corresponding to haplotypes in the haploblock Ch3B_B9 on chromosome 3B. (**d**) Haplotype block based on two SNP markers on chromosome 3B; haplotype significantly associated with the Type_2_2019&2020 trait (MTA) marked with red. Frequency shows occurrence (percentage) of haplotypes in the group of families.

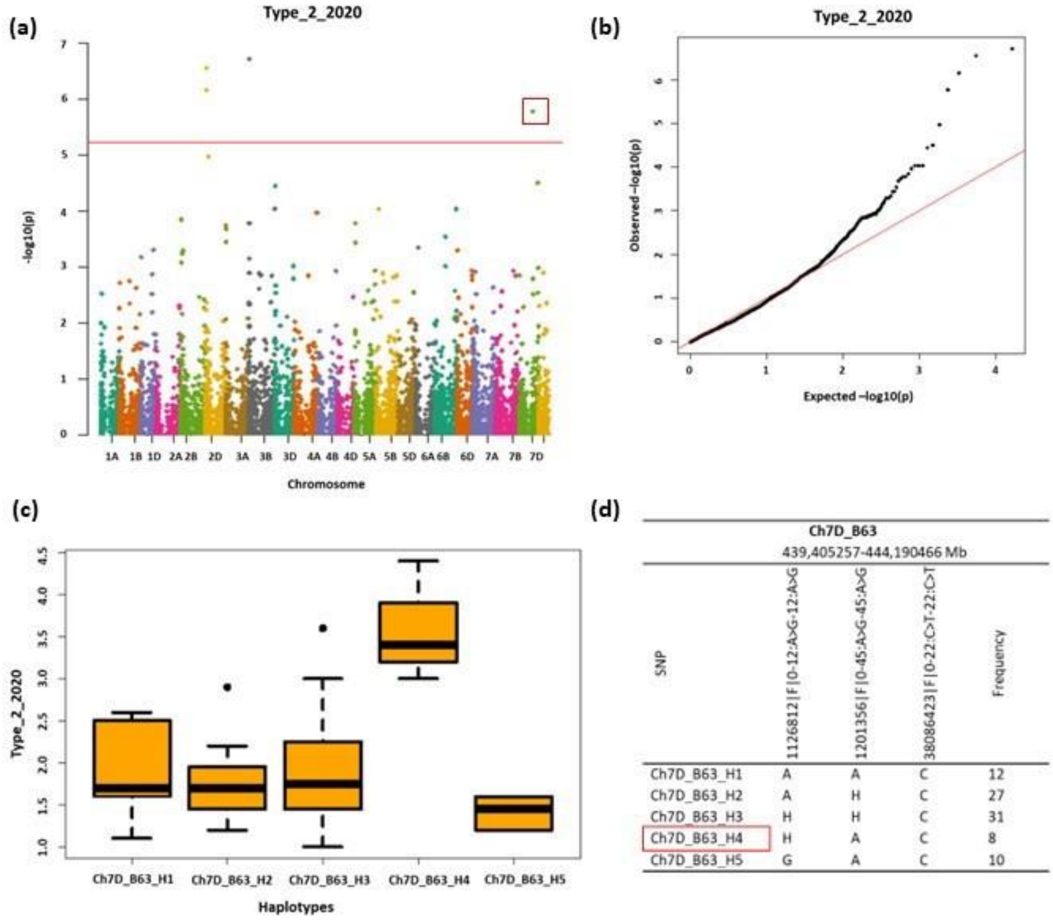

Ch7D_B63 439,405257-444,190466 Mb				
SNP	1126812|F|0-12:A>G-12:A>G	1201356|F|0-45:A>G-45:A>G	38086423|F|0-22:C>T-22:C>T	Frequency
Ch7D_B63_H1	A	A	C	12
Ch7D_B63_H2	A	H	C	27
Ch7D_B63_H3	H	H	C	31
Ch7D_B63_H4	H	A	C	8
Ch7D_B63_H5	G	A	C	10

Figure S6. (**a**) Manhattan plot on 21 wheat chromosomes for Type_2_2020 trait. Red horizontal dash represents the GWAS threshold of -log(p) = 5.22 for Bonferroni correction and dots above the red line indicate significant MTAs. (**b**) Q-Q plot for GWAS results for Type_2_2020 trait. (**c**) Boxplots indicating the phenotype value of Type_2_2020 trait corresponding to haplotypes in the haploblock Ch7D_B63 on chromosome 7D. (**d**) Haplotype block based on three SNP markers on chromosome 7D; haplotype significantly associated with the Type_2_2020 trait (MTA) marked with red. Frequency shows occurrence (percentage) of haplotypes in the group of families.
