# Supplementary material for: Evaluation of Fusarium Head Blight Resistance Effects by Haplotype-Based Genome-Wide Association Study in Winter Wheat Lines Derived by Marker Backcrossing Approach
Source: Int J Mol Sci. 2022 Nov 17;23(22):14233. doi: 10.3390/ijms232214233 (PMC9695032; doi:10.3390/ijms232214233)
Supplement: Supplementary file 1 [file ijms-23-14233-s001.zip › Tables S1-S4.pdf]

Table S1. Phenotypic data lines/cultivars tested with two isolates of *F. culmorum.*

No	Line/Cultivar	Group	Type_1_2019	Type_2_2019	Type_1&2_2019	Type_1_2020	Type_2_2020	Type_1&2_2020	Type_1_2019&2020	Type_2_2019&2020	Type_1&2_2019&2020	FHBi
1	Belenus	Wheat cultivars	1.4	3.6	2.5	2.0	1.6	1.8	1.7	2.6	2.2	80
2	Belissa	Wheat cultivars	1.6	2.4	2.0	1.6	2.0	1.8	1.6	2.2	1.9	80
3	Błyskawica	Wheat cultivars	1.8	4.4	3.1	1.8	1.9	1.9	1.8	3.2	2.5	90
4	Ceres (durum)	Wheat cultivars	1.8	5.8	3.8	1.0	3.3	2.2	1.4	4.6	3.0	60
5	Euforia	Wheat cultivars	2.0	3.4	2.7	1.8	2.0	1.9	1.9	2.7	2.3	70
6	Opcja	Wheat cultivars	1.8	2.8	2.3	1.4	1.8	1.6	1.6	2.3	2.0	70
7	Plejada	Wheat cultivars	1.8	4.2	3.0	1.2	2.9	2.1	1.5	3.6	2.5	60
8	Reduta	Wheat cultivars	2.4	2.4	2.4	1.4	5.4	3.4	1.9	3.9	2.9	70
9	Sfera	Wheat cultivars	1.8	3.4	2.6	1.4	1.8	1.6	1.6	2.6	2.1	90
10	Tobak	Wheat cultivars	1.6	5.2	3.4	2.2	3.4	2.8	1.9	4.3	3.1	90
11	Wilejka	Wheat cultivars	2.0	2.6	2.3	2.0	3.3	2.7	2.0	3.0	2.5	70
12	FUS_12_52	Family Fhb1	1.4	1.4	1.4	2.0	1.1	1.6	1.7	1.3	1.5	20
13	FUS_12_76	Family Fhb1	1.8	1.2	1.5	1.8	1.3	1.6	1.8	1.3	1.5	30
14	FUS_24_130	Family Fhb1	1.0	1.6	1.3	1.6	1.6	1.6	1.3	1.6	1.5	20
15	FUS_24_131	Family Fhb1	1.6	2.6	2.1	1.4	1.9	1.7	1.5	2.3	1.9	40
16	FUS_24_133	Family Fhb1	1.8	1.6	1.7	1.2	2.2	1.7	1.5	1.9	1.7	60
17	FUS_24_134	Family Fhb1	1.0	1.8	1.4	1.2	1.5	1.4	1.1	1.7	1.4	20
18	FUS_24_140	Family Fhb1	1.2	1.8	1.5	1.8	2.0	1.9	1.5	1.9	1.7	40
19	FUS_24_169	Family Fhb1	1.4	1.6	1.5	1.4	1.7	1.6	1.4	1.7	1.5	40
20	FUS_27_241	Family Fhb1	1.4	1.6	1.5	2.6	1.4	2.0	2.0	1.5	1.8	30
21	FUS_27_243	Family Fhb1	1.2	1.2	1.2	1.4	1.4	1.4	1.3	1.3	1.3	20
22	FUS_27_246	Family Fhb1	1.8	2.0	1.9	2.4	1.6	2.0	2.1	1.8	2.0	30
23	FUS_27_247	Family Fhb1	1.8	2.2	2.0	1.4	1.5	1.5	1.6	1.9	1.7	20
24	FUS_27_249	Family Fhb1	1.8	1.6	1.7	1.6	1.3	1.5	1.7	1.5	1.6	20
25	FUS_27_255	Family Fhb1	1.8	2.0	1.9	1.4	1.3	1.4	1.6	1.7	1.6	30
26	FUS_27_280	Family Fhb1	1.4	2.0	1.7	1.4	1.8	1.6	1.4	1.9	1.7	10
27	FUS_27_292	Family Fhb1	2.2	1.4	1.8	2.8	1.4	2.1	2.5	1.4	2.0	20
28	FUS_27_296	Family Fhb1	1.8	1.6	1.7	1.2	1.1	1.2	1.5	1.4	1.4	30
29	FUS_27_301	Family Fhb1	2.2	1.8	2.0	1.8	1.2	1.5	2.0	1.5	1.8	40
30	FUS_27_302	Family Fhb1	2.0	1.8	1.9	2.2	1.5	1.9	2.1	1.7	1.9	40
31	FUS_27_305	Family Fhb1	1.6	2.2	1.9	1.6	1.6	1.6	1.6	1.9	1.8	40
32	FUS_27_316	Family Fhb1	2.4	1.4	1.9	2.4	1.2	1.8	2.4	1.3	1.9	30
33	FUS_27_318	Family Fhb1	1.8	1.0	1.4	1.6	1.3	1.5	1.7	1.2	1.4	30
34	FUS_27_343	Family Fhb1	2.2	1.8	2.0	1.8	1.4	1.6	2.0	1.6	1.8	50
35	FUS_27_1369	Family Fhb1	2.2	1.0	1.6	1.2	1.2	1.2	1.7	1.1	1.4	40
36	FUS_34_369	Family Fhb1	1.4	1.6	1.5	1.6	1.7	1.7	1.5	1.7	1.6	60
37	FUS_34_383	Family Fhb1	1.2	1.4	1.3	2.0	1.5	1.8	1.6	1.5	1.5	70
38	FUS_34_395	Family Fhb1	1.4	3.2	2.3	1.8	1.5	1.7	1.6	2.4	2.0	60

39	FUS_34_403	Family Fhb1	1.2	1.8	1.5	2.2	1.1	1.7	1.7	1.5	1.6	50
40	FUS_34_405	Family Fhb1	1.4	1.6	1.5	1.6	1.2	1.4	1.5	1.4	1.5	50
41	FUS_34_420	Family Fhb1	1.2	1.6	1.4	1.4	1.4	1.4	1.3	1.5	1.4	40
42	FUS_34_429	Family Fhb1	2.0	1.6	1.8	1.8	1.6	1.7	1.9	1.6	1.8	60
43	FUS_34_441	Family Fhb1	1.6	1.8	1.7	1.2	1.5	1.4	1.4	1.7	1.5	70
44	FUS_34_458	Family Fhb1	1.2	1.8	1.5	1.6	1.2	1.4	1.4	1.5	1.5	60
45	FUS_34_468	Family Fhb1	2.2	1.4	1.8	1.8	1.6	1.7	2.0	1.5	1.8	70
46	FUS_34_470	Family Fhb1	1.4	1.8	1.6	2.2	1.0	1.6	1.8	1.4	1.6	40
47	FUS_34_475	Family Fhb1	1.4	1.6	1.5	1.8	1.9	1.9	1.6	1.8	1.7	50
48	FUS_34_1495	Family Fhb1	1.4	1.4	1.4	1.8	1.6	1.7	1.6	1.5	1.6	60
49	FUS_40_481	Family Fhb1	1.2	2.0	1.6	1.0	1.8	1.4	1.1	1.9	1.5	40
50	FUS_40_484	Family Fhb1	1.8	1.8	1.8	2.0	1.5	1.8	1.9	1.7	1.8	60
51	FUS_40_491	Family Fhb1	2.0	1.4	1.7	1.2	1.7	1.5	1.6	1.6	1.6	40
52	FUS_40_494	Family Fhb1	1.6	2.2	1.9	1.4	1.5	1.5	1.5	1.9	1.7	60
53	FUS_40_495	Family Fhb1	1.8	1.6	1.7	2.2	1.3	1.8	2.0	1.5	1.7	60
54	FUS_40_498	Family Fhb1	1.8	2.2	2.0	2.4	1.8	2.1	2.1	2.0	2.1	80
55	FUS_40_506	Family Fhb1	1.6	1.8	1.7	1.8	1.8	1.8	1.7	1.8	1.8	80
56	FUS_40_510	Family Fhb1	2.4	2.2	2.3	1.8	1.7	1.8	2.1	2.0	2.0	70
57	FUS_40_511	Family Fhb1	1.8	2.4	2.1	1.4	1.5	1.5	1.6	2.0	1.8	60
58	FUS_40_513	Family Fhb1	1.2	1.8	1.5	1.4	1.7	1.6	1.3	1.8	1.5	60
59	FUS_40_516	Family Fhb1	1.4	1.4	1.4	1.6	1.7	1.7	1.5	1.6	1.5	50
60	FUS_40_529	Family Fhb1	1.6	1.2	1.4	1.4	1.9	1.7	1.5	1.6	1.5	40
61	FUS_12_10_c	Check family	2.0	2.6	2.3	1.8	2.5	2.2	1.9	2.6	2.2	30
62	FUS_12_16_c	Check family	1.8	2.8	2.3	1.8	2.3	2.1	1.8	2.6	2.2	30
63	FUS_12_27_c	Check family	2.0	2.6	2.3	2.0	2.6	2.3	2.0	2.6	2.3	40
64	FUS_24_147_c	Check family	1.2	2.8	2.0	1.0	2.9	2.0	1.1	2.9	2.0	60
65	FUS_24_150_c	Check family	1.4	2.6	2.0	1.6	3.6	2.6	1.5	3.1	2.3	30
66	FUS_24_153_c	Check family	1.4	3.2	2.3	1.8	3.0	2.4	1.6	3.1	2.4	60
67	FUS_27_248_c	Check family	1.8	1.4	1.6	2.2	2.9	2.6	2.0	2.2	2.1	50
68	FUS_27_268_c	Check family	1.6	2.8	2.2	1.4	2.2	1.8	1.5	2.5	2.0	60
69	FUS_27_269_c	Check family	1.6	2.8	2.2	1.4	2.0	1.7	1.5	2.4	2.0	60
70	FUS_34_381_c	Check family	1.8	3.6	2.7	1.2	3.0	2.1	1.5	3.3	2.4	80
71	FUS_34_389_c	Check family	1.4	2.0	1.7	2.2	3.4	2.8	1.8	2.7	2.3	70
72	FUS_34_390_c	Check family	1.6	2.8	2.2	1.8	4.4	3.1	1.7	3.6	2.7	80
73	FUS_40_482_c	Check family	1.6	3.0	2.3	1.6	2.9	2.3	1.6	3.0	2.3	80
74	FUS_40_483_c	Check family	1.6	2.0	1.8	2.0	2.3	2.2	1.8	2.2	2.0	80
75	FUS_40_502_c	Check family	1.4	3.2	2.3	1.2	2.2	1.7	1.3	2.7	2.0	90
76	SMH 8694 (S)	Susceptible checks	1.8	3.0	2.4	2.8	5.4	4.1	2.3	4.2	3.3	90
77	SMH 8816 (S)	Susceptible checks	1.4	3.6	2.5	1.4	5.2	3.3	1.4	4.4	2.9	80
78	DL325/11/3 (S)	Susceptible checks	1.6	4.0	2.8	1.6	6.2	3.9	1.6	5.1	3.4	90
79	KBP 14 16 (S)	Susceptible checks	1.4	4.6	3.0	1.6	6.2	3.9	1.5	5.4	3.5	100
80	20828 [Fhb1-]	Resistant checks	1.4	1.0	1.2	1.0	1.2	1.1	1.2	1.1	1.2	30
81	A40-19-1-2	Resistant checks	1.2	1.4	1.3	1.4	1.1	1.3	1.3	1.3	1.3	40
82	Arina	Resistant checks	1.8	1.8	1.8	1.6	2.2	1.9	1.7	2.0	1.9	80
83	Fregata	Resistant checks	2.4	1.6	2.0	1.0	2.1	1.6	1.7	1.9	1.8	50
84	UNG 136.6.1.1 [Fhb1+]	Resistant checks	2.0	1.6	1.8	1.4	1.1	1.3	1.7	1.4	1.5	70

85	S 10 [Fhb1+]	Resistant checks	1.6	1.2	1.4	2.4	1.0	1.7	2.0	1.1	1.6	30
86	S 30 [Fhb1+]	Resistant checks	1.2	0.6	0.9	1.8	1.4	1.6	1.5	1.0	1.2	40
87	S 32 [Fhb1+]	Resistant checks	1.8	0.8	1.3	2.0	0.9	1.4	1.9	0.8	1.4	40

Table S2. Haplotypes described in MTA with Bonferroni correction represented in wheat families.

**HT**	**Wheat families**
Ch2D_B35_H1	FUS_12_52, FUS_12_76, FUS_24_130, FUS_24_131, FUS_24_134, FUS_24_140, FUS_24_169, FUS_27_243, FUS_27_301, FUS_27_246, FUS_27_280, FUS_27_249, FUS_27_255, FUS_27_292, FUS_34_1495, FUS_34_369, FUS_34_458, FUS_34_420, FUS_34_429, FUS_34_395, FUS_34_405, FUS_34_468, FUS_34_381_c, FUS_40_513, FUS_40_481, FUS_40_516, FUS_40_484, FUS_40_491, FUS_40_495, FUS_40_510, FUS_40_494, FUS_34_470
Ch2D_B35_H2	FUS_12_16_c, FUS_12_27_c, FUS_12_10_c, FUS_24_150_c, FUS_24_153_c, FUS_27_247, FUS_27_269_c, FUS_27_268_c, FUS_27_248_c, FUS_34_389_c, FUS_34_390_c, FUS_40_511, FUS_40_483_c, FUS_40_502_c, FUS_40_482_c
Ch3B_B9_H2	FUS_12_27_c, FUS_12_10_c, FUS_24_150_c, FUS_24_153_c, FUS_24_147_c, FUS_27_247, FUS_27_269_c, FUS_27_268_c, FUS_27_248_c, FUS_34_389_c, FUS_34_390_c, FUS_40_511, FUS_40_483_c, FUS_40_502_c, FUS_40_482_c
Ch7D_B63_H4	FUS_34_381_c, FUS_34_389_c, FUS_34_390_c

Table S3. Haploblocks used for association weight matrix creation.

**No**	**Haploblock**	**Chromosome**	**Start (bp[1])**	**Stop (bp)**	**Influence[2]**	**No of connections[3]**
1	Ch1B_B57	1B	550476176	554267102	0	9
2	Ch6D_B99	6D	493598133	493701062	0	18
3	Ch7A_B103	7A	663484676	672234914	0	17
4	Ch2D_B35	2D	32388539	32388595	0	15
5	Ch5A_B45	5A	461525128	478177901	0	4
6	Ch6D_B91	6D	485009388	485216394	0.3	15
7	Ch5D_B25	5D	301099862	306943258	1.1	15
8	Ch2D_B44	2D	40479955	45844025	1.3	11
9	Ch2D_B65	2D	146980860	146981379	1.3	14
10	Ch3B_B11	3B	15056092	16649400	1.5	16
11	Ch3A_B6	3A	12622011	12622051	1.7	16
12	Ch3A_B8	3A	13838412	13838478	1.7	16
13	Ch3B_B6	3B	10276362	10999531	1.7	16
14	Ch3B_B9	3B	13276831	13633016	1.7	16
15	Ch2B_B17	2B	29914867	30019767	2.3	17
16	Ch1B_B82	1B	648260077	649478384	3.3	18
17	Ch5D_B80	5D	541495332	546776260	4.1	17
18	Ch3D_B5	3B	6675747	7017502	4.1	14
19	Ch3B_B12	3B	16819271	16819271	5.9	15

[1] base pairs; [2] according to betweenness centrality metrics; [3] according to degree centrality metrics; [2] and [3] calculated according to OpenOrd protocol in Gephi 0.9.2 tool.

Table S4. Polish winter wheat breeding lines tested as potential recurrent parent in comparison to *Fhb1* gene donor line AIII62. Microsatellite allele products.

				Marker name (product size- bp)			
**No**	**Name**	**Origin**	**UMN10**	**gwm389**	**barc12**	**gwm493**	**gpw3248**
1	AIII62*	Radzików	239	139	187	205	254
2	Zawisza	HR Smolice[1]	236	139	187	144	250
**3**	**SMH 8527**	**HR Smolice**	**236**	**151**	**177**	**144**	**269**
4	SMH 113	HR Smolice	236	143	187	144	271
5	SMH 213	HR Smolice	236	u	177	144	264
6	SMH 8553	HR Smolice	236	139	u	144	269
7	SMH 8823	HR Smolice	236	137	221	144	271
8	DD 13 /10-4	Danko[2] HR	236	121	177	144	250
9	DD 237/09	Danko HR	236	u	198	144	264
10	DD 283/10	Danko HR	236	u	187	144	250
11	DC 489/10	Danko HR	236	121	u	144	250
12	DC 4010/10	Danko HR	236	143	187	u	250
13	DC 4176/09	Danko HR	236	121	177	144	254
14	DL 338/10	Danko HR	236	143	187	u	254
**15**	**DL 414/10**	**Danko HR**	**236**	**121**	**253**	**144**	**u**
16	DL 428/10	Danko HR	236	114	177	147	254
17	STH 6212	HR Strzelce[3]	236	u	214	144	250
**18**	**STH 1178**	**HR Strzelce**	**236**	**143**	**198**	**144**	**271**
19	STH 1116	HR Strzelce	236	u	198	144	250
20	STH 3099	HR Strzelce	236	u	177	144	254
21	STH 10- 0007	HR Strzelce	236	143	198	144	250
22	STH 10- 0010	HR Strzelce	236	143	187	144	250
23	STH 11637	HR Strzelce	236	139	177	200	252
**24**	**MIB 11 262**	**Małopolska[4] HR**	**236**	**151**	**177**	**144**	**250**
25	MIB 12 221	Małopolska HR	236	139	221	144	254
26	AND 990/09	Poznańska[5] HR	236	u	177	144	252
27	AND 462/09	Poznańska HR	236	121	187	144	252
28	AND 242/10	Poznańska HR	236	u	187	u	261
29	AND 708/10	Poznańska HR	236	u	177	144	269
**30**	**NAD 10041**	**Poznańska HR**	**236**	**151**	**177**	**144**	**269**
31	NAD 11053	Poznańska HR	236	139	187	144	271
32	NAD 8046	Poznańska HR	236	u	187	u	250
33	NAD 10080	Poznańska HR	236	u	177	144	264
34	KBP 10-18 A	Małopolska HR	236	137	198	147	254
35	KBP 10-8	Małopolska HR	236	139	177	144	264

Polish breeding company [1]Hodowla Roślin Smolice, Smolice, Poland; [2]Danko Hodowla Roślin, Choryń, Poland; [3]Hodowla Roślin Strzelce, Strzelce, Poland; [4]Małopolska Hodowla Roślin, Kraków Poland; [5]Poznańska Hodowla Roślin, Tulce, Poland;

* indicates *Fhb1* donor line derived in Plant Breeding and Acclimatization Institute – National Research Institute, Radzików, Poland

- bold indicates breeding lines chosen as a RP,
- u - unknown PCR product, bp – base pairs
